# Inhibition of multidrug resistance protein 1 (MRP1) improves chemotherapy drug response in primary and recurrent glioblastoma multiforme

**DOI:** 10.3389/fnins.2015.00218

**Published:** 2015-06-16

**Authors:** Amanda Tivnan, Zaitun Zakaria, Caitrín O'Leary, Donat Kögel, Jenny L. Pokorny, Jann N. Sarkaria, Jochen H. M. Prehn

**Affiliations:** ^1^Department of Physiology and Medical Physics, Centre for Systems Medicine, Royal College of Surgeons in IrelandDublin, Ireland; ^2^Experimental Neurosurgery, Neuroscience Center, Frankfurt University HospitalFrankfurt am Main, Germany; ^3^Department of Radiation Oncology, Mayo ClinicRochester, MN, USA

**Keywords:** glioblastoma, multidrug resistance protein 1, siRNA, chemoresistance

## Abstract

Glioblastoma multiforme (GBM) is a highly aggressive brain cancer with extremely poor prognostic outcome despite intensive treatment. All chemotherapeutic agents currently used have no greater than 30–40% response rate, many fall into the range of 10–20%, with delivery across the blood brain barrier (BBB) or chemoresistance contributing to the extremely poor outcomes despite treatment. Increased expression of the multidrug resistance protein 1(MRP1) in high grade glioma, and it's role in BBB active transport, highlights this member of the ABC transporter family as a target for improving drug responses in GBM. In this study we show that small molecule inhibitors and gene silencing of MRP1 had a significant effect on GBM cell response to temozolomide (150 μM), vincristine (100 nM), and etoposide (2 μM). Pre-treatment with Reversan (inhibitor of MRP1 and P-glycoprotein) led to a significantly improved response to cell death in the presence of all three chemotherapeutics, in both primary and recurrent GBM cells. The presence of MK571 (inhibitor of MRP1 and multidrug resistance protein 4 (MRP4) led to an enhanced effect of vincristine and etoposide in reducing cell viability over a 72 h period. Specific MRP1 inhibition led to a significant increase in vincristine and etoposide-induced cell death in all three cell lines assessed. Treatment with MK571, or specific MRP1 knockdown, did not have any effect on temozolomide drug response in these cells. These findings have significant implications in providing researchers an opportunity to improve currently used chemotherapeutics for the initial treatment of primary GBM, and improved treatment for recurrent GBM patients.

## Introduction

Glioblastoma multiforme (GBM) is a highly aggressive grade IV brain cancer with an extremely poor prognostic outcome despite intensive treatment regimes. GBM represent approximately 17% of all primary brain tumors diagnosed worldwide; and 60–75% of astrocytomas, increasing in frequency with age (WHO and IARC, 2008). Prognosis is reported as “median survival” which, for adults with aggressive GBM treated according to the standard Stupp protocol (Stupp et al., 2005) is 14.6 months. The average five year survival is less than 3%, leading to the fact that GBM is the most lethal form of brain tumor.

The standard clinical treatment of newly diagnosed GBM is known as the Stupp protocol, outlined in 2005 (Stupp et al., 2005) as radiotherapy (fractionated focal irradiation in daily fractions of 2 Gy given for 5 days per week for 6 weeks for a total of 60 Gy) in combination with daily temozolomide (TMZ) (75 mg/m^2^ body surface area) administered continuously. Such protocols increased median survival rates in GBM patients from 12.1 months with RT alone to 14.6 months for TMZ/RT treatment.

Despite surgical resection of GBM tumors, recurrence at distal sites is typically 6.9 months (Stupp et al., 2005, 2009, 2010) and in instances where repeat resection is not a viable option, adjunct chemotherapy is ineffective at stopping tumor progression and eventually, morbidity. Chemotherapy used for recurrent GBM includes the PCV regime [procarbazine, CCNU (lomustine), and vincristine] (Brada et al., 2010) and/or the ACE regime (bevacizumab combined with carboplatin and etoposide); both of which are used as palliative therapy in recurrent GBM. All agents used; however, have no greater than 30–40% response rate and many fall into the range of 10–20%(Bota et al., 2007) with delivery or chemoresistance contributing to the extremely poor patient outcomes despite treatment.

A major hindrance to several chemotherapeutic agents in effective GBM treatment is their efficient transport across the blood brain barrier (BBB). For this reason, several researchers have focused their studies on novel mechanisms of drug delivery across the BBB and distribution throughout the brain (Campbell et al., 2008). The role of the BBB in brain homeostasis is maintained through the action of active efflux transporters of the ATP-binding cassette (ABC) family including p-glycoprotein (Pgp), the multidrug resistance proteins (MRPs), and breast cancer resistance protein (BCRP) (Loscher and Potschka, 2005a,b,c). The significant role which MRPs play in chemoresistant GBM patients was identified in 1995 by Abe et al. (1994) with high grade gliomas (HGGs) showing a significant increase in expression of several MRPs including MRP1 (ABCC1). However, it was the localized expression of MRP1 within GBM tumor specimens themselves in addition to the tumor vasculature, identified by Calatozzolo et al. (2005) which was of particular interest to the authors. As such, this paper outlines the role of MRP1 inhibition in improving chemotherapy drug response in both primary and recurrent GBM patient-biopsy derived cell lines; as evaluated *in vitro*, suggesting an intrinsic chemoresistance role of MRP1 expression in GBM tumor cells, independent of the B-BB endothelial transport system. In this regard, it may be suggested that increased MRP1 expression in high grade gliomas, such as glioblastoma multiforme, contribute to chemoresistance through increased drug efflux and reduced bioavailability of the administered chemotherapeutic within the cancerous cell. Improving intracellular exposure to efficient chemotherapeutics, through MRP1 targeted reduction, would significantly increase GBM cell death when used in combination with chemotherapeutic agents.

## Materials and methods

### Cell culture and siRNA transfections

Commercially available glioblastoma cell lines U251 and A172 (ECACC), glioblastoma cell lines derived from primary tumors, MZ-327 and MZ-18 and glioblastoma cell lines derived from recurrent grade IV tumors, MZ-256 and MZ-304 (Hetschko et al., 2008), were grown as a monolayer in DMEM with 10% heat-inactivated fetal calf serum, 100 U/ml penicillin, and 100 mg/ml streptomycin and maintained in a humidified incubator at 37°C and 5% CO_2_. For siRNA transfections, cells were seeded at 7 × 10^5^ cells in a T25 flask (GIBCO) and maintained at 37°C and 5% CO_2_ for 24 h. Media was then removed from all flasks in the dark, and replaced with 5 ml of OptiMem media (GIBCO, UK). Cells were transiently transfected with either MRP1-specific siRNA (GATGACACCTCTCAACAAAdTdT 30 nM) or a non-targeting negative control siRNA (30 nM, ON-TARGET plus Non-targeting siRNA#1, Dharmacon, US), using Lipofectamine 2000 (Invitrogen, US) according to manufacturer's protocol. All flasks were incubated at 37°C and 5% CO2 for 96 h.

### Protein extraction and western blot analysis

Transfected cells were washed with phosphate buffered saline (PBS), trypsinised and cells were centrifuged at 1000 rpm for 3 min. The supernatant was then removed and protein was extracted from cells by resuspending the resulting pellet in RIPA buffer (50 mM Tris pH 7.4, 150 mM NaCl, 0.2% NP-40, 50 mM NaF, 5 mM EDTA, 0.1 mM orthovanadate, plus protease inhibitor cocktail SIGMA [P8340]). Resuspended cells in RIPA were left on ice for 15 min before centrifugation at 4°C and 16,000 rpm for 15 min. Protein supernatant was taken from each sample, quantified and stored at −20°C. Patient-derived primary glioblastoma (G6, G8, G12, G38, G39, G43, G44, G59, and G75), recurrent glioblastoma (G14, G46, G64, and G76), Oligoastrocytoma (G10), and Gliosarcoma (G22 and G28) lysates were provided by Mayo Clinic Brain Tumor SPORE (Giannini et al., 2005; Sarkaria et al., 2006, 2007, 2008).

### Western blotting

Western blot analysis was performed on lysates prepared as outlined previously. Western blots were carried out using 4–10% gradient pre-cast gels and HEPES running buffer (PIERCE). Protein samples were mixed with 10 μl of 1xDTT loading buffer (6x buffer: 4x Tris.Cl/SDS pH6.8, Glycerol 30%, SDS 10%, DTT 0.6 M, Bromophenol Blue 0.012%) and loaded into gels. Gels were run at 100 V for 45 min and transferred onto nitrocellulose membranes at 45 V for 90 min, using wet transfer buffer [10x Transfer buffer (100 ml), Methanol (200 ml), sterile water (700 ml)] [10xTranfers buffer: Tris (30.3 g), glycine (144 g) Sterile H_2_O (1000 ml)]. Membranes were then blocked in 10%-milk-TBST overnight at 4°C. Membranes were incubated with primary anti-MRP1 antibody (Enzo Life Sciences ALX-801-007-C125; 1:500) in TBST-milk (0.5%) for 2 h at room temperature. Membranes were then washed three times with TBST for 5 min and secondary anti-rat HRP conjugated antibody (Abcam, 1:5000) was then added for a further 2 h. Loading control protein beta actin was assessed by incubation of the membrane with anti-beta actin antibody (Abcam, 1:5000) in TBST for 1 h at room temperature. Membranes were then washed in TBST three times for 5 min each, after which a secondary anti-mouse HRP conjugated antibody (Sigma, 1:5000) was added for 1 h, followed by further washes with TBST (3 × 5 min). All segments were then developed and the images assessed on a LAS Imager 3000. Densitometry analysis of protein bands was analyzed using ImageJ, MRP1 protein bands were normalized to B-actin loading controls and unpaired Student *T*-tests were carried out where appropriate.

### Drug treatment and Metylthiazol Tetrazolium assay (MTT) assay

Glioblastoma cell lines were seeded into a 96 well plate either directly, or 6 h post transfection with an MRP1-targeting or a non-targeting negative control siRNA. Cells were seeded onto 96 well plates at a concentration of 1 × 10^3^ cells per well and incubated for 72 h at 37°C and 5% CO2 to allow MRP1 messenger RNA suppression to occur. Cells were then treated with either control media or one of three chemotherapy drugs temozolomide (150 μM), vincristine (100 nM), or etoposide (2 μM) (generously provided by Dr. Markus Rehm). Cells were then returned to the incubator for a further 72 h; after which time, Metylthiazol Tetrazolium (MTT) powder in PBS (50 μl of 5 mg/ml) was added to each well. Cells were then incubated for a further 4 h after which all solution was removed and dimethyl sulfoxide (DMSO) was added. After 10 min incubation time at 37°C, absorbance was recorded at 570 nm wavelength and data was recorded and analyzed. Small molecule inhibitors MK571 (25 μM) and Reversan (15 μM) were added 7 h prior to carrying out further drug treatment (temozolomide, vincristine or etoposide) or assay assessment (media change for proliferation and 2D-migration assays).

### Cell proliferation assay

Glioblastoma cells U251, MZ-256, and MZ-327 were pre-treated with the MRP1 small molecule inhibitor MK571 (M7571 SIGMA) and Reversan (SML0173 SIGMA) at a final concentration of 25 and 15 μM; respectively, for 7 h and subsequently were seeded at 1 × 10^4^ cells/well in a 6 well plate and allowed to adhere overnight. Cells were then allowed to grow for 96 h and counted using the trypan blue exclusion method.

### Cell migration assay

Glioblastoma cells U251, MZ-256, and MZ-327 were pre-treated with the MRP1 small molecule inhibitor Reversan (SML0173 SIGMA) at a final concentration of 15 μM for 7 h and subsequently seeded at 5 × 10^4^ cells into wound chambers (Ibidi, US, Cat#80206) and allowed to adhere overnight. The wound assay insert was removed, the initial wound was photographed, cells were allowed to grow for 19–24 h, dependent on cell type, and then wound closure was re-photographed and assessed using WimScratch Quantitative Wound Healing Image Analysis (Ibidi, USA).

## Results

### GBM cell lines drug response after small molecule inhibition of MRP1 using MK571 and Reversan

**Figure 1:**
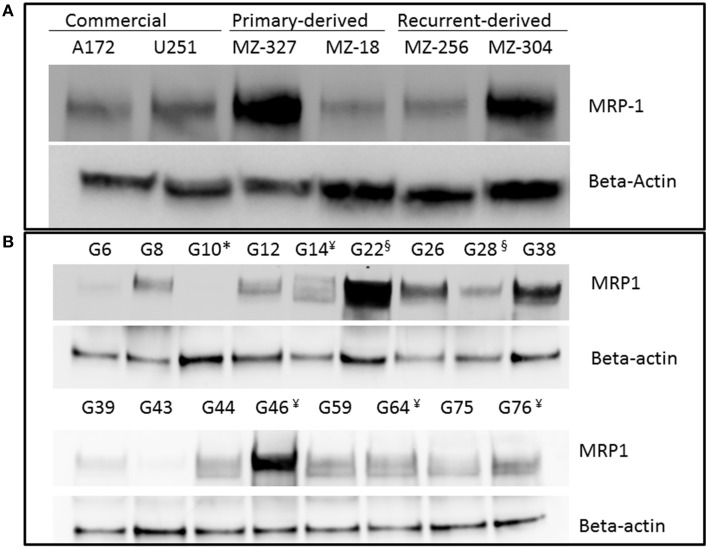
**Western Blot of MRP1 expression in commercial, primary, and recurrent GBM cell lines**. Western blot analysis was performed on lysates prepared from A172, U251, MZ-327, MZ-18, MZ-256 and MZ-304 glioblastoma cell lines **(A)** Additionally, patient-derived primary glioblastoma (G6, G8, G12, G38, G39, G43, G44, G59, and G75), recurrent glioblastoma¥ (G14, G46, G64, and G76), Oligoastrocytoma^*^ (G10), and Gliosarcoma^§^ (G22 and G28) lysates were assessed for MRP1 expression **(B)** Developed membranes showed MRP1 expression at 190 kDa in varying amounts across the cell lines assessed.

**Figure 2:**
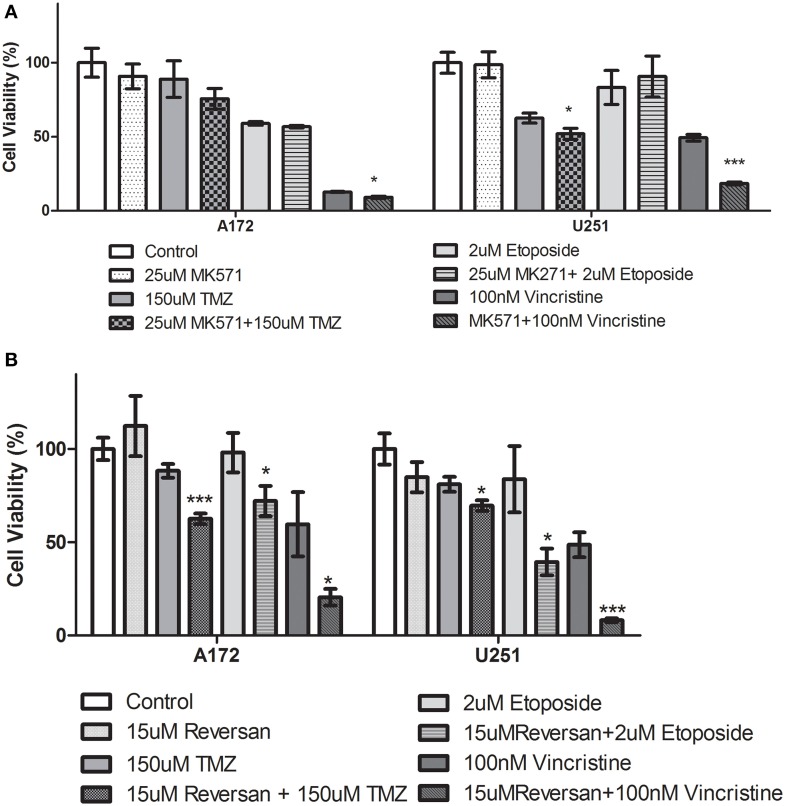
**Small Molecule inhibition of MRP1 and chemoresponse in GBM Commercial cell lines. (A)** The effects of MK571 on temozolomide, vincristine or etoposide-induced cell death in commercial GBM lines is negligible. MK571 enhanced vincristine-induced cell death (^*^p < 0.05) in A172 cells while treatment of U251 cells with the small molecule MRP1 inhibitor leads to enhanced temozolomide and vincristine-induced cell death relative to chemotherapy alone treatment (^*^p < 0.05). Treatment of A172 and U251 with Reversan (15 μM) leads to a significant increase in temozolomide, vincristine, and etoposide-induced cell death compared to chemotherapy drug treatment alone **(B)** (*n* = 3 ^*^p < 0.05, ^***^p < 0.001, Unpaired Student *T*-test).

**Figure 3:**
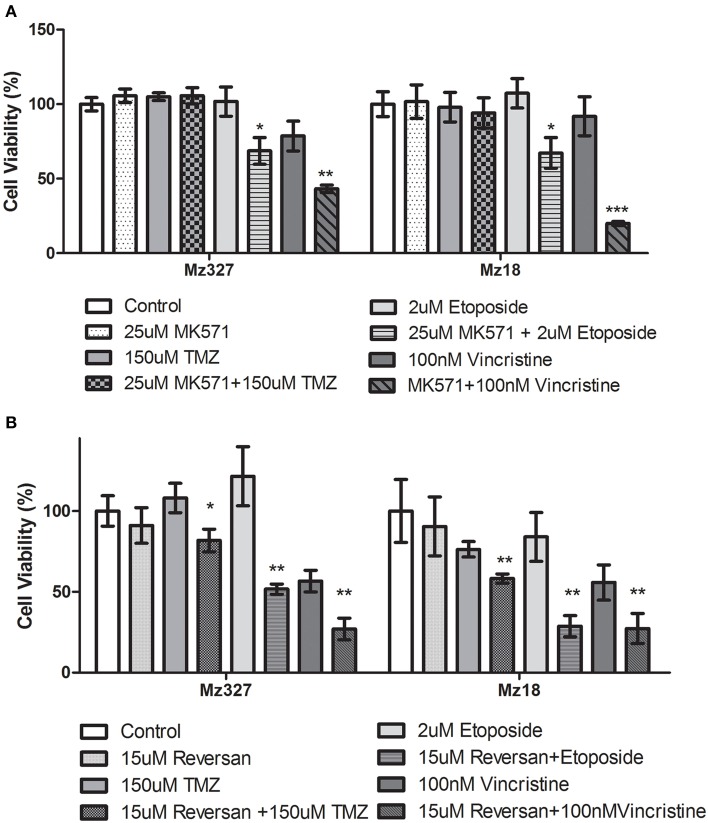
**Small Molecule inhibition of MRP1 and chemoresponse in Primary GBM tumor derived cell lines. (A)** Treatment of primary tumor-derived GBM cell lines, MZ-327, and MZ-18, led to a significant increase in both vincristine and etoposide-induced cell death compared to chemotherapy treatment alone (*n* = 3 ^*^p < 0.05, ^**^p < 0.01, ^***^p < 0.001 Unpaired Student *T*-test). The presence of MK571 (25 μM) did not have any effect on temozolomide–induced cell death in either of these cell lines. Treatment with Reversan (15 μM) significantly increased cell death for all three chemotherapies tested (*n* = 3 ^*^p < 0.05, ^**^p < 0.01 Unpaired Student *T*-test) in both lines assessed **(B)** relative to single chemotherapy drug treatment.

**Figure 4:**
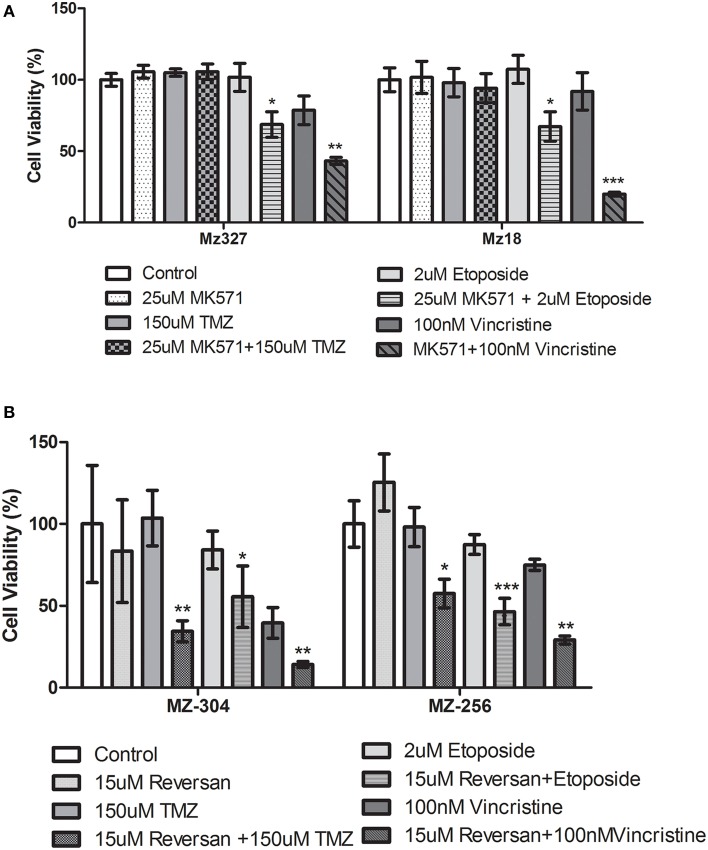
**Small molecule inhibition of MRP1 and chemoresponse in Recurrent GBM tumor derived cell lines**. Treatment of recurrent tumor-derived GBM cell lines, MZ-304, and MZ-256, led to a significant increase in both vincristine and etoposide-induced cell death (*n* = 3 ^*^p < 0.05, ^**^p<0.01, ^***^p < 0.001 Unpaired Student *T*-test) **(A)** compared to chemotherapy alone treatment. The presence of MK571 (25 μM) did not have any effect on temozolomide-induced cell death in either of these cell lines. Treatment with Reversan (15 μM), significantly increased cell death for all three chemotherapies tested (*n* = 3 ^*^p < 0.05, ^**^p < 0.01, ^***^p < 0.001 Unpaired Student *T*-test) in both lines assessed **(B)**.

A variety of glioblastoma multiforme cell lines were chosen for this study based upon their origins and available clinical data. Commercially available GBM cell lines A172 and U251, cell lines derived from primary GBM tumor biopsies (designated MZ-327 and MZ-18), along with cells established from a recurrent tumor biopsy (MZ-256 and MZ-304) (Hetschko et al., 2008; Murphy et al., 2014) were used in assessing the effects of pre-treating GBM cells with MRP1 small molecule inhibitors. All cell lines were assessed in terms of their MRP1 protein expression when grown as a monolayer in DMEM media (Figure 1A). Additionally, patient-derived primary glioblastoma (G6, G8, G12, G38, G39, G43, G44, G59, and G75), recurrent glioblastoma (G14, G46, G64, and G76), Oligoastrocytoma (G10), and Gliosarcoma (G22 and G28) lysates were assessed with respect to their MRP1 expression (Figure 1B). To allow analysis across various cell lines, densitometry analysis of protein bands was carried out using ImageJ, MRP1 protein bands were normalized to B-actin loading controls (Supplementary Figures 1A,B). In terms of *in vitro* assessment, A172, U251, MZ-327, MZ-18, MZ-256, and MZ-304 were used for evaluation of MRP1 inhibition and chemotherapy response. Addition of small molecule inhibitors of MRP1 had a significant effect on GBM cell drug responses to temozolomide, vincristine, and etoposide. Notably, MK571 is a non-specific inhibitor of MRP1, also known to act on MRP4 (Reid et al., 2003); while Reversan not only inhibits MRP1, but also P-glycoprotein (Pgp) very effectively (Burkhart et al., 2009; Henderson et al., 2011). As shown in Figure 2A, inhibition of MRP1 and MRP4 by MK571 did not lead to a profound change in drug-induced cell death in any of the commercial cell lines assessed. Pre-treatment with Reversan however, which would inhibit MRP1 and Pgp, led to an improved response in terms of temozolomide, vincristine and etoposide-induced cell death, Figure 2B. The most notably enhanced cell death was evident in both A172 and U251 cells treated with a combination of Reversan and vincristine (100 nm). In the case of primary (MZ-327 and MZ-18, Figure 3A) and recurrent (MZ-256 and MZ-304, Figure 4A) GBM tumor biopsy derived cell lines; in both cases the presence of MK571 led to an enhanced effect of vincristine and etoposide in reducing cell viability over a 72 h period. MRP1 and MRP4 inhibition by MK571 did not have any effect on temozolomide drug response in these cells. Reversan-mediated inhibition of MRP1 and Pgp led to significant enhancement of temozolomide, vincristine and etoposide-induced cell death in primary (Figure 3B) and recurrent (Figure 4B) GBM cell lines.

### Specific inhibition of MRP1 using short-interfering (si)RNA and drug response assessment

**Figure 5:**
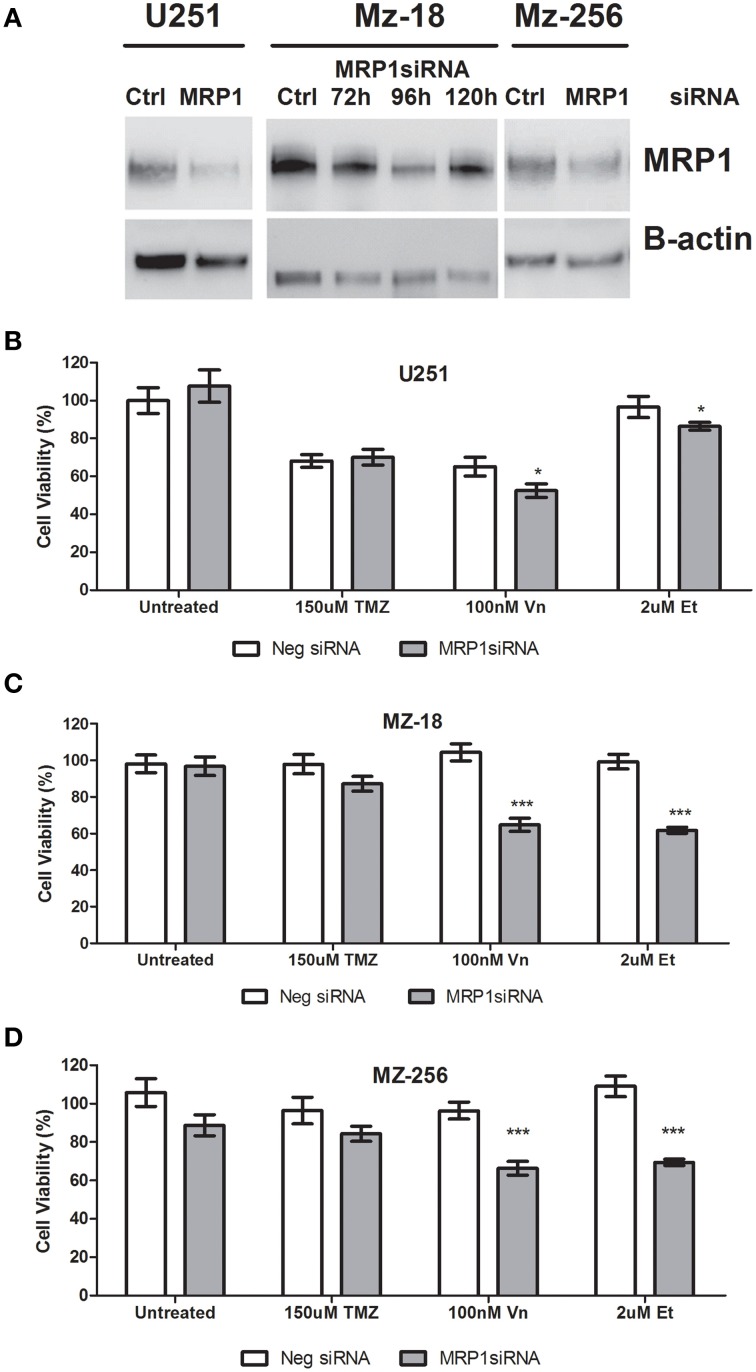
**Evaluation of specific MRP1 inhibition using siRNA and the effect of MRP1 knockdown on chemotherapy-induced cell death**. Glioblastoma cell lines; U251, MZ-18, and MZ-256, representing commercial, primary tumor derived and recurrent tumor derived glioblastoma cell lines were transiently transfected with MRP1siRNA. MRP1 protein (190 kDa band) was reduced in all three cell lines 96 h post-transfection **(A)**. *Lane 1*: Negative control siRNA-treated U251, *Lane 2*: MRP1siRNA-treated U251, *Lane 3*: Negative control siRNA-treated MZ-18, *Lane 4*: MRP1 siRNA-treated MZ-18 after 72 h, *Lane 5*: MRP1 siRNA-treated MZ-18 after 96 h, *Lane 6*: MRP1 siRNA-treated MZ-18 after 120 h, *Lane 7*: Negative control siRNA-treated MZ-256, *Lane 8*: MRP1 siRNA-treated MZ-256 after 96 h. The effect of specific MRP1 inhibition, using siRNA, was evaluated for temozolomide (150 μM), vincristine (100 nM), or etoposide (2 μM)– induced cell death and a significant increase in vincristine and etoposide-induced cell death in all three cell lines assessed; commercial **(B)**, primary **(C)**, and recurrent **(D)**-tumor derived representatives (*n* = 3 ^*^p < 0.05, ^***^p < 0.001 Unpaired Student *T*-test) relative to single chemotherapy-induced cell death was noted.

As mentioned previously, due to the non-specific nature of the small molecule inhibitors currently available to assess MRP1 inhibition *in vitro*, siRNA were designed which specifically target MRP1 mRNA; hereby inhibiting protein expression. As shown in Figure 5A, transient transfection of U251, MZ-18, and MZ-256 cells led to reduced MRP1 expression after 96 h. Notably, as shown in lanes 3, 4, 5, and 6 of Figure 5A; MRP1 expression post-transfection is optimal at 96 h, with a noted re-expression of the target protein by 120 h. This was confirmed using densitometry analysis of MRP1 protein bands, analyzed using ImageJ, where MRP1 protein was normalized to B-actin loading controls and unpaired Student *T*-tests were carried out to verify target suppression relative to negative control siRNA treated cell lysates (Supplementary Figure 1C). In this regard, all further experiments required siRNA treatment to be carried out, a minimum of, 72 h prior to drug treatment, thereby ensuring reducing MRP1 expression prior to drug administration. Figures 5B–D, depict the data obtained from MTT assays of commercial (U251), primary (MZ-18), and recurrent (MZ-256) GBM established cell lines. As can be seen in these figures, specific MRP1siRNA inhibition leads to a significant reduction in cell viability in all three GBM cell lines when combined with vincristine (100 nM) or etoposide (2 μM). There was no noted change in response to temozolomide (150 μM) under these conditions, indicating that any effect noted previously in response to Reversan pre-treatment may be attributed to Pgp inhibition and not MRP1 targeting. Notably, all statistical analysis of control vs. treatment; or single vs. combination treatment groups is represented in Supplementary Table 2.

### Assessment of alternative role of MRP1 inhibition in GBM

**Figure 6:**
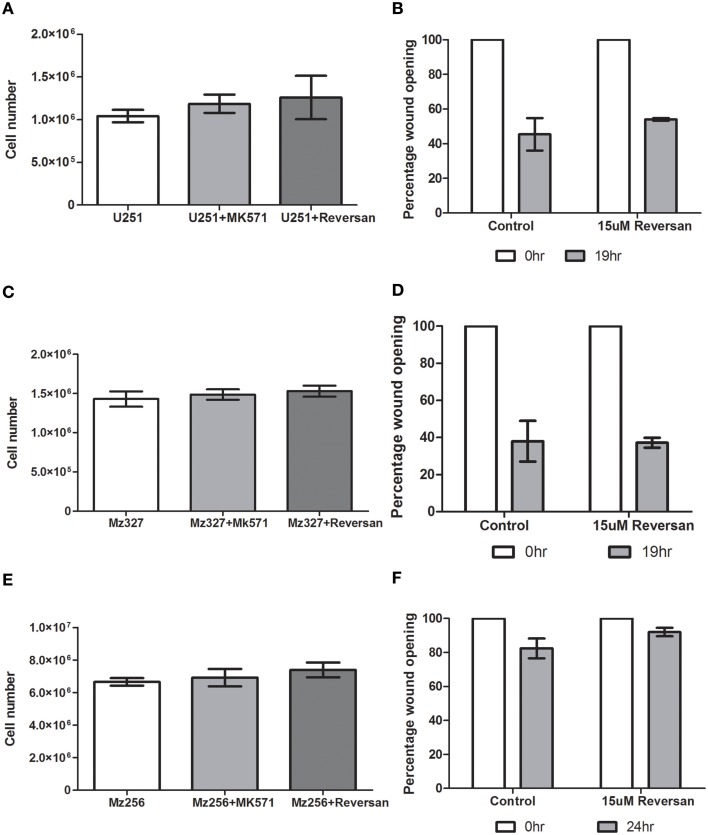
**The effect of MK571 and Reversan on cell proliferation and 2D-migration in commercial, primary, and recurrent GBM cell lines**. Glioblastoma cells U251 **(A)**, MZ-327 **(C)**, and MZ-256 **(E)** were pre-incubated with non-specific MRP1 small molecule inhibitors MK571 (25 μM) or Reversan (15 μM) and proliferation and wound-closure capabilities assessed. No significant change in proliferation was noted in any of the representative cell lines assessed. Similarly, treatment of glioblastoma cell lines with Reversan does not alter cell motility in commercial **(B)**, primary **(D)** and recurrent **(F)** GBM cells.

Based on previous findings in neuroblastoma cells (Burkhart et al., 2009; Henderson et al., 2011), the effect of MK571 and Reversan treatment of GBM cells was assessed *in vitro*. As depicted in Figures 6A,C,E, there was no alteration in cell proliferation rates between controls and treated commercial, primary or recurrent GBM tumor derived lines over a 72 h period. Additionally, as reduced cell motility was noted in neuroblastoma cells after exposure to Reversan and with MRP1siRNA transfection (Henderson et al., 2011), a 2D-migration or wound closure assay, was carried out 7 h after pre-treatment of the cells with this small molecule inhibitor (15 μM, Figures 6B,D,F). In the case of glioblastoma cells however, Reversan treatment did not lead to any change in wound closure abilities in commercial, primary or recurrent GBM cell lines.

## Discussion

Glioblastoma multiforme is the most aggressive form of brain cancer with a current median survival post-diagnosis of 14.6 months when treated. The Stupp protocol involves surgery and concurrent Temozolomide administration with radiotherapy (Stupp et al., 2005). A major challenge in terms of effective treatment of brain tumors is penetration of the blood brain barrier (BBB), a highly selective permeability barrier that separates the circulating blood from the brain extracellular fluid in the central nervous system (CNS). The physiology of this barrier is highly researched in an attempt to provide a means of ensuring active drug transport and efficient drug delivery to target regions of the brain. The role of MRP1, along with other membrane transporters including Pgp, in BBB function includes the efflux of cytotoxic hydrophobic drugs (Regina et al., 1998). In addition, the fact that MRP1 has been shown to be highly expressed in high grade glioma patient samples (Benyahia et al., 2004), with localization of MRP1 to the luminal side of brain capillary endothelial cells (Nies et al., 2004) suggests that the efficient efflux action of MRP1 in the GBM tumor microenvironment may contribute to the highly resistant nature of GBM tumors to current chemotherapeutics. This makes inhibition of MRP1 an attractive approach to improve drug influx to both GBM tumor brain regions and within brain tumor cells.

This study was carried out on a collection of both primary and recurrent patient-derived GBM tumor biopsy cell lines in addition to the commercially available U251 and A172 lines with the aim of identifying the true potential which MRP1 inhibition may play in primary or recurrent GBM tumor treatment. Of initial interest was the expression levels of MRP1 protein in the panel of GBM lines intended for *in vitro* study and also patient-derived tumor lysates from primary and recurrent glioblastoma. As a point of interest, a single oligoastrocytoma and two gliosarcoma patient tumor-derived lysates were also included in Western blotting analysis. Notably, the variable expression noted between individual samples (Figure 1), despite commonality in tumour stage, in addition to the varied genetic characteristics of each patient tumor sample (Supplemental Table 1) highlights the heterogeneity of this disease and the need for a more personalized and direct treatment approach as opposed to the pan cellular treatment currently employed through use of chemotherapy.

In addition to evaluating the effects of reduced MRP1 expression on drug resistance in GBM cells, the possibility of MRP1 playing an alternative role in GBM tumor formation was also evaluated. Previous findings in neuroblastoma cells (Burkhart et al., 2009; Henderson et al., 2011) showed that Reversan was capable of reducing cell motility and colony formation devoid of any effect on proliferation rates. In this regard, we assessed whether non-specific MRP1 inhibition by Reversan and/or MK571 led to any alteration in wound closure or proliferation rates in glioblastoma cells. Unlike the case for neuroblastoma, MRP1 inhibition in this study did not appear to play any role in cell migration or growth, independent of its role in drug resistance in GBM cells.

The most notable, and clinically relevant, finding presented in this publication, through the use of the MRP1 and MRP4 small molecule inhibitor, MK571, and also an MRP1 specific siRNA, is that MRP1 inhibition enhanced Vincristine- and Etoposide-, but not Temozolomide-induced cell death in primary or recurrent GBM cell lines. Inhibition of MRP1 and Pgp, using Reversan (15 μM), and subsequent treatment with temozolomide (150 μM) however led to a statistically significant increase in cell death compared to temozolomide treatment alone across all primary, recurrent and commercial cell lines assessed in this study. In 2013, Veringa et al. (2013) detailed a list of substrate specificity for classical therapeutics for a range of efflux transporters, including MRP1 and Pgp. In their findings they list that temozolomide is a substrate for Pgp and Breast cancer related protein (BCRP) but not MRP1. Additionally, our findings corroborate Peignan et al. (2011), who note a lack of effect on cell death in the commercial GBM line T98G when MRP1 siRNA was used *in vitro*.

Vincristine and etoposide are two additional chemotherapies which are currently used in recurrent GBM treatment regimens as a means of palliative care, therefore the clinical application of assessing their improved efficacy was of great interest in both primary and recurrent GBM cell lines. MRP1 inhibition using MK571, Reversan and MRP1siRNA led to a significant enhancement in the cell death capabilities of both vincristine (100 nM) and etoposide (2 μM). In terms of MK571 use, neither etoposide nor vincristine are MRP4 substrates (Dallas et al., 2004), however both are known to be MRP1 substrates (Veringa et al., 2013). This, in addition to the use of an MRP1-specific siRNA devoid of off-target effects proves that the specific inhibition of the MRP1 transporter protein allows both vincristine (100 nM) and etoposide (2 μM) to induce cell death more effectively in GBM cells *in vitro*.

The findings of this study have significant implications in terms of providing researchers an opportunity to improve currently used chemotherapeutics for the initial treatment of primary GBM, and improved treatment for recurrent GBM patients. Additionally, the data obtained during this study is highly significant for further *in vivo* assessment of GBM orthotopic murine models of chemoresistance.

## Funding

This work is funded by the Irish Cancer Society Research Fellowship CRF13TIV, awarded to AT, supported by Tesco Charity of the Year.

### Conflict of interest statement

The authors declare that the research was conducted in the absence of any commercial or financial relationships that could be construed as a potential conflict of interest.
