# Supplementary material for: Inhibition of multidrug resistance protein 1 (MRP1) improves chemotherapy drug response in primary and recurrent glioblastoma multiforme
Source: Front Neurosci. 2015 Jun 16;9:218. doi: 10.3389/fnins.2015.00218 (PMC4468867; doi:10.3389/fnins.2015.00218)
Supplement: Supplementary file 1 [file Table1.PDF]

Inhibition of Multidrug resistance protein 1 (MRP1) improves chemotherapy drug response in primary and recurrent glioblastoma multiforme

Amanda Tivnan.[1], Zaitun Zakaria[1], Caitrín O'Leary[1], Donat Kögel[2], Jenny L. Pokorny [3], Jann N. Sarkaria[3], Jochen H.M. Prehn[1]

Supplementary Table 1 Clinical Data for Glioma Patient-derived Tumour Lysates. All data provided courtesy of Mayo Clinic Brain Tumor SPORE

Sample	Human Subject Age	Human Subject Gender	Human Subject Diagnosis	MGMT Methylation status	MGMT Mutation	EGFR AMPLIFI-CATION	PTEN	P53 MUTATION	V600E BRAF mutation	p16 deleti-on
G6	65.1	M	GBM PRIMARY	Unmethyl.	WT	Y	W	R273C	N	Y
G8	74.7	F	GBM PRIMARY	Methyl.	WT	Y	HD	W	N	N
G12	68.6	M	GBM PRIMARY	Methyl	WT	Y	W	SPLICE	N	Y
G26	48.6	M	GBM PRIMARY	Unmethyl	250: Leu>Leu	Y	HD	W	N	Y
G38	71.7	F	GBM PRIMARY	Unmethyl	WT	Y	W	R110C		Y
G39	51	M	GBM PRIMARY	Methyl	WT	Y	W	W	N	Y
G43	69.4	M	GBM PRIMARY	Unmethyl	WT	N	W	F270C	N	Y
G44	79.5	F	GBM PRIMARY	Unmethyl	WT	N	W	W	Y	Y
G59	82.9	F	GBM PRIMARY	Methyl		Y	HD	W	N	Y
G75	62.2	F	GBM PRIMARY	Unmethyl	250: Leu>Leu	Y				
G14	57.8	M	GBM RECURRENT	Unmethyl	WT	N	INDEL		N	Y
G46	55.8	M	GBM RECURRENT	Methyl	WT	Y	W	Q353K	N	
G64	64.1	F	GBM RECURRENT	Unmethyl	WT	Y		W		
G76	38.3	M	GBM RECURRENT	Methyl	WT			W		
G10	41.5	M	OLIGO-ASTROCYTOMA	Unmethyl	WT	N	HD	W	N	Y
G22	80.1	M	GLIOSARCOMA	Methyl	625: Lys>Arg	N	W	R273C	N	Y
G28	67.6	M	GLIOSARCOMA	Unmethyl	WT	N	G132D	M246T	N	N
