# Supplementary material for: Inhibition of multidrug resistance protein 1 (MRP1) improves chemotherapy drug response in primary and recurrent glioblastoma multiforme
Source: Front Neurosci. 2015 Jun 16;9:218. doi: 10.3389/fnins.2015.00218 (PMC4468867; doi:10.3389/fnins.2015.00218)
Supplement: Supplementary file 2 [file Table2.DOCX]

Supplementary Table 2

**P values of treatment groups as depicted in figures 2, 3 and 4. Unpaired Student T-test.**

|  | **A172** | **U251** |
| --- | --- | --- |
| **Control vs MK571** | 0.49 | 0.903 |
| **Control vs TMZ** | 0.055 | 9.24x10^-5^ |
| **Control vs Et** | 0.007 | 0.21 |
| **Control vs Vn** | 7.7x10^-6^ | 1.07x10^-6^ |
| **Control vs Reversan** | 0.25 | 0.1 |
| **MK571+TMZ vs TMZ** | 0.18 | 0.025 |
| **MK571+Vincristine vs Vincristine** | 0.004 | 9.2x10^-9^ |
| **MK571+Etoposide vs Etoposide** | 0.08 | 0.35 |
| **Reversan+TMZ vs TMZ** | 0.0001 | 0.02 |
| **Reversan+Vincristine vs Vincristine** | 0.03 | 0.001 |
| **Reversan+ Etoposide vs Etoposide** | 0.04 | 0.04 |
|  |  |  |
|  | **MZ-327** | **MZ-18** |
| **Control vs MK571** | 0.384 | 0.879 |
| **Control vs TMZ** | 0.347 | 0.664 |
| **Control vs Et** | 0.872 | 0.0329 |
| **Control vs Vn** | 0.038 | 2.55x10^-6^ |
| **Control vs Reversan** | 0.548 | 0.728 |
| **MK571+TMZ vs TMZ** | 0.46 | 0.4 |
| **MK571+Vincristine vs Vincristine** | 0.002 | 0.0001 |
| **MK571+Etoposide vs Etoposide** | 0.02 | 0.0003 |
| **Reversan+TMZ vs TMZ** | 0.023 | 0.004 |
| **Reversan+Vincristine vs Vincristine** | 0.005 | 0.0032 |
| **Reversan+ Etoposide vs Etoposide** | 0.002 | 0.004 |
|  |  |  |
|  | **MZ-256** | **MZ-304** |
| **Control vs MK571** | 0.879 | 0.119 |
| **Control vs TMZ** | 0.875 | 0.727 |
| **Control vs Et** | 0.815 | 0.182 |
| **Control vs Vn** | 0.936 | 1.03x10^-9^ |
| **Control vs Reversan** | 0.285 | 0.734 |
| **MK571+TMZ vs TMZ** | 0.22 | 0.25 |
| **MK571+Vincristine vs Vincristine** | 0.0002 | 0.001 |
| **MK571+Etoposide vs Etoposide** | 0.001 | 0.0263 |
| **Reversan+TMZ vs TMZ** | 0.03 | 0.01 |
| **Reversan+Vincristine vs Vincristine** | 3.46E-07 | 0.01 |
| **Reversan+ Etoposide vs Etoposide** | 0.001 | 0.04 |
