# Supplementary figures and images for: Inhibition of multidrug resistance protein 1 (MRP1) improves chemotherapy drug response in primary and recurrent glioblastoma multiforme
Source: Front Neurosci. 2015 Jun 16;9:218. doi: 10.3389/fnins.2015.00218 (PMC4468867; doi:10.3389/fnins.2015.00218)

A

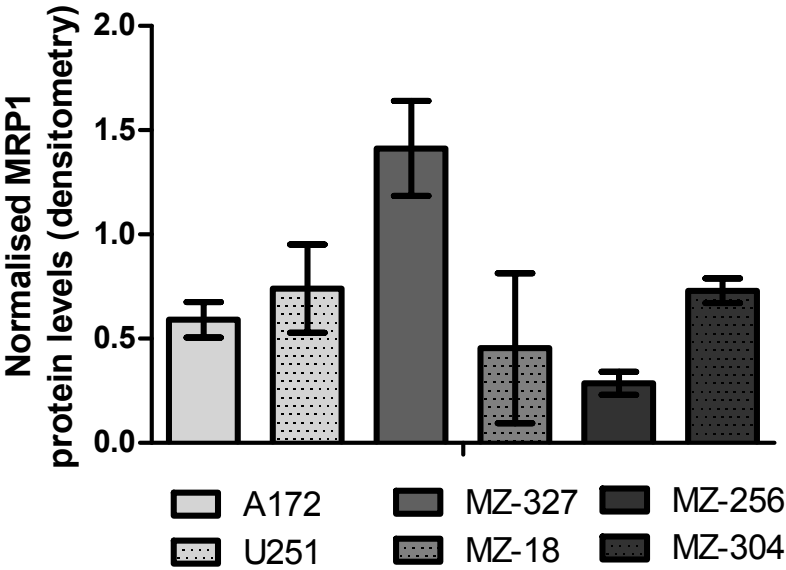

B

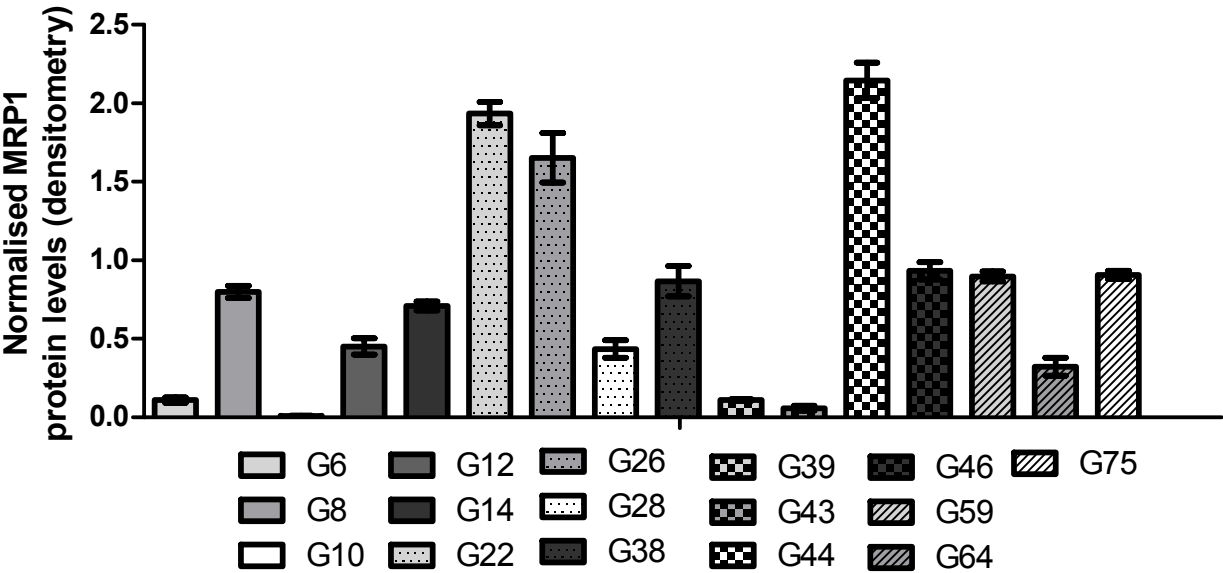

C

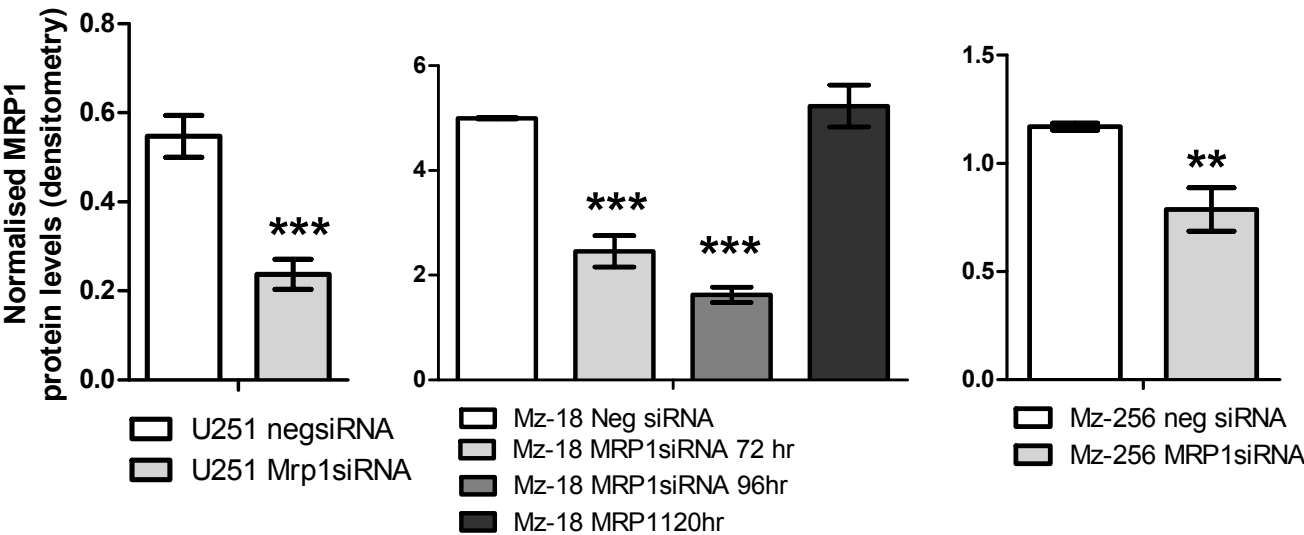

Supplement: Supplementary file 3 [file Image1.PDF]
